# Integrating Telemedicine in Botulinum Toxin Type-A Treatment for Spasticity Management: Perspectives and Challenges from Italian Healthcare Professionals

**DOI:** 10.3390/toxins16120529

**Published:** 2024-12-07

**Authors:** Stefania Spina, Salvatore Facciorusso, Nicoletta Cinone, Luigi Santoro, Anna Castagna, Marina Ramella, Franco Molteni, Andrea Santamato, The Collaborative Working Group

**Affiliations:** 1 Spasticity and Movement Disorders “ReSTaRt”, Physical Medicine and Rehabilitation Section, Department of Medical and Surgical Sciences, University of Foggia, 71122 Foggia, Italy; spinastefania.ss@gmail.com (S.S.);; 2 IRCCS Fondazione Don Carlo Gnocchi, 20148 Milan, Italy; 3 Villa Beretta Rehabilitation Center, Valduce Hospital Como, 23845 Costa Masnaga, Italy

**Keywords:** telemedicine, spasticity, usability, hybrid care model, botulinum toxin Type-A

## Abstract

(1) Background: Telemedicine is a vital tool for enhancing healthcare accessibility and outcomes at reduced costs. This study aimed to assess the usability of the Maia Connected Care telemedicine platform for managing spasticity in patients receiving botulinum toxin type-A, focusing on the perspectives of Italian physiatrists with expertise in this treatment. (2) Methods: Conducted from March 2023 to June 2023, this multicenter survey involved 15 Italian physicians who used the platform for teleconsultations. Data collected included demographic details, responses to the Telemedicine Usability Questionnaire, and physician insights on patient satisfaction, treatment effectiveness, and implementation challenges in telehealth. (3) Results: The platform demonstrated high usability, with strong physician satisfaction due to its user-friendly interface and quality of interactions. A majority expressed willingness to continue telehealth for spasticity management, noting its effectiveness in improving patient satisfaction and outcomes. Challenges included replicating the depth of in-person consultations and addressing issues like reimbursement and telehealth standardization. (4) Conclusions: This study highlights telemedicine’s potential for spasticity management and clinician satisfaction, while underscoring the need for improvements in simulating in-person experiences and addressing systemic issues. The absence of patient perspectives represents a limitation, advocating for future research to optimize telemedicine practices and evaluate long-term clinical impacts.

## 1. Introduction

Telemedicine is the use of technology to provide clinical healthcare services to patients at a distance [1]. It is a subset of telehealth, which encompasses all healthcare services that are delivered through technology. Telemedicine has found its application across a broad range of medical specialties [2,3] and patient demographics, revolutionizing the accessibility and delivery of healthcare services. It offers the potential to enhance access to care, improve health outcomes, and reduce healthcare costs [4].

In recent years, telemedicine has been increasingly adopted in physical medicine and rehabilitation [5]. Telemedicine holds immense potential for physiatry, particularly in expanding care access for individuals living with disabilities, neurological disorders, and mobility issues, as well as those managing chronic conditions. The conventional demand for physical presence at appointments, high frequency of healthcare service utilization, need for specialized medical attention, and coordination across different care disciplines could all be substantially mitigated with telemedicine, providing a more patient-centered and accessible healthcare approach.

One area where telemedicine has shown promise is in the management of spasticity, a common condition seen in patients with neurological disorders such as stroke, multiple sclerosis, spinal cord injury and cerebral palsy. Spasticity is characterized by velocity-dependent increase in muscle tone, resulting in stiffness and resistance to movement [6]. Its significant impact on patient function, quality of life, and caregiver burden [7] necessitates complex management strategies, including pharmacological interventions, physical therapy, and assistive devices [8]. Among the pharmacological treatments, botulinum toxin type-A (BoNT-A) has been recognized as an effective intervention for focal spasticity, requiring careful monitoring and follow-up care [9]. Traditional in-person follow-up presents several challenges in BoNT-A treatment management. These include scheduling constraints, travel burden for patients with mobility limitations, and the need for frequent assessments to optimize treatment timing and dosing. The COVID-19 pandemic particularly highlighted telemedicine’s capacity to maintain essential healthcare delivery while ensuring safety through social distancing [10].

The patient-centered approach crucial for spasticity management aligns well with telemedicine’s capabilities. Regular monitoring of treatment responses, goal assessment, and therapy adjustments can potentially be facilitated through remote consultations. However, despite these apparent advantages, telemedicine adoption in spasticity management remains in its early stages [11,12], and there is a need for more research to understand its effectiveness, usability, and challenges. Understanding healthcare providers’ perceptions and experiences with telemedicine is crucial for successful implementation [13].

In Italy, telemedicine development has followed a structured pathway. The 2014 “National Guidelines for Telemedicine” aimed to standardize initiatives nationwide, while the 2020 “National Directives for the Delivery of Telemedicine Services” established regulatory frameworks equating telemedicine with traditional care within the National Health Service. The National Recovery and Resilience Plan (2021–2026) allocated €1 billion for telemedicine projects [14]. However, despite this policy progress, actual adoption of telemedicine remains fragmented and limited. A 2022 survey of over 700 Italian healthcare professionals found only 13% were currently using telemedicine [15]. Key barriers included lack of training, equipment, and conviction about its usefulness. Regarding specific applications, telemedicine is being used in Italy for specialist teleconsultations, remote monitoring of chronic conditions like diabetes and heart disease, and providing care continuity for elderly and frail patients at home [16]. Despite the overall growth of telemedicine in Italy, there is limited information on its specific application in spasticity treatment, highlighting the need for focused studies.

The rapid adoption of telemedicine has revealed significant knowledge gaps in its application to specialized medical fields. Particularly in spasticity management, the current literature lacks a comprehensive understanding of telemedicine platform usability and its practical implementation. While telemedicine platforms have proliferated across various medical specialties, their specific application in spasticity management remains poorly documented, especially regarding healthcare professionals’ experiences and perspectives. Furthermore, within the Italian healthcare system, there exists a notable absence of systematic evaluation regarding implementation challenges and outcomes in this specialized field.

To address these knowledge gaps, our research pursued several interconnected objectives. The primary aim was to evaluate the usability of the Maia Connected Care tele-medicine platform, specifically focusing on its application by Italian physiatrists experienced in BoNT-A administration for spasticity management. Through this evaluation, we sought to generate comprehensive insights into the platform’s effectiveness and practical utility in clinical settings. Secondarily, we aimed to identify and analyze the challenges and barriers that healthcare providers encounter when adopting telemedicine for spasticity management, thereby providing valuable insights for future implementation strategies.

This study also focused on assessing healthcare provider satisfaction and their perceptions of treatment effectiveness, recognizing that provider acceptance and confidence are crucial determinants of successful telemedicine integration. Additionally, we sought to document specific patterns of platform utilization and various applications within spasticity management, creating a foundation for establishing best practices and standardized protocols in this emerging field of telemedicine application.

## 2. Results

### 2.1. Participants

The study sample consisted of 15 physiatrists, with a gender distribution of 60% female (*n* = 9) and 40% male (*n* = 6). The age of the participants ranged from 35 to 66 years, reflecting a broad spectrum of career stages and clinical experience. 

Between March and June 2023, participating physicians conducted 450 total teleconsultations. The distribution of visit types showed 120 first visits (26.7%) and 330 follow-up visits (73.3%). Treatment modifications were documented across several categories: dosage adjustments were implemented in 185 cases, exercise program modifications in 210 cases, and equipment adaptations in 95 cases. Referral to in-person care was necessary in 45 cases, primarily due to complex assessment requirements or technical limitations. In cases requiring detailed physical examination, local healthcare providers (physical therapists or nurses) were present with the patient in 25% of teleconsultations, while family caregivers assisted in 15% of cases, particularly during functional assessments and demonstration of exercise programs.

Detailed characteristic and professional data for the participants are listed in Table 1.

### 2.2. Telemedicine Usability Assessment

The mean total score across all items was 116.20 (9.13) (max score 147), indicating a generally positive perception towards telehealth. The mean scores for each item on the TUQ (Telemedicine Usability Questionnaire) were calculated, providing an overall measure of the platform’s usability from the perspective of the physicians (Table 2). 

The usefulness domain achieved a mean score of 16.07 (SD = 2.15) out of 21. Participants particularly endorsed the platform’s ability to improve service delivery (mean = 5.87, SD = 0.99) and time efficiency (mean = 5.27, SD = 0.59). The ease-of-use domain demonstrated strong results, with participants reporting high scores for system simplicity (mean = 6.40, SD = 0.63) and learnability (mean = 6.47, SD = 0.74).

The Quality Interface quality evaluation yielded positive results across all metrics. Participants rated the platform’s pleasantness of interaction (mean = 5.80, SD = 0.77) and system comprehensibility (mean = 6.40, SD = 0.63) favorably. Interaction quality scores revealed strong performance in audio clarity (mean = 5.93, SD = 0.70) and communication effectiveness (mean = 6.07, SD = 0.80). However, visual interaction capabilities received lower ratings (mean = 3.73, SD = 1.44).

System reliability assessment revealed some limitations in comparison to traditional in-person visits (mean = 3.13, SD = 1.30). However, participants reported satisfactory experiences with error recovery (mean = 5.07, SD = 0.80) and system guidance for problem resolution (mean = 4.87, SD = 1.19).

Overall satisfaction with the telemedicine system was high (mean = 6.33, SD = 0.62). Participants expressed strong comfort levels with telehealth communication (mean = 6.00, SD = 0.93) and indicated robust intentions for continued use (mean = 6.47, SD = 0.52).

**Figure 1:**
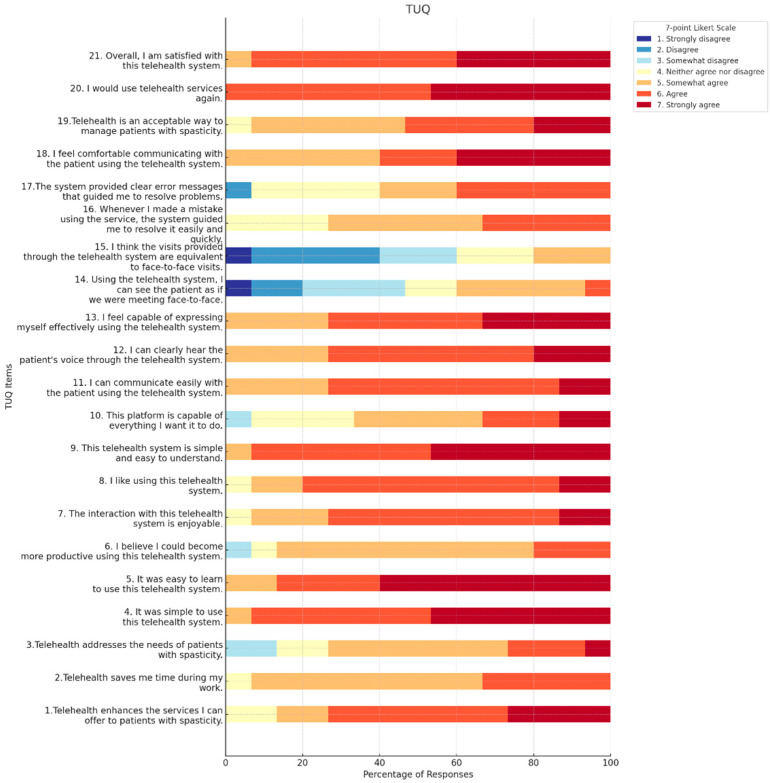
Stacked bar chart telemedicine usability questionnaire items.

The bar plot in Figure 1 provides a visual representation of the participants’ responses for the 21-item.

### 2.3. Expert Opinion

Physician assessment of patient satisfaction with telehealth visits was notably positive (mean = 5.93, SD = 0.59). The reliability of rehabilitation goals established during telehealth sessions received favorable ratings (mean = 5.67, SD = 0.82). Post-infiltration assessment capabilities demonstrated effectiveness in both verifying injection site accuracy (mean = 5.40, SD = 0.63) and facilitating dosage adjustment decisions (mean = 5.60, SD = 0.74).

### 2.4. Challenges and Issue

**Figure 2:**
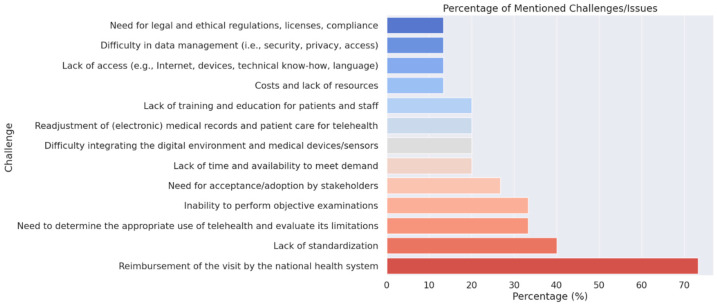
Bar plot of challenges and issues to face in telehealth.

Analysis of implementation barriers revealed several priority areas for improvement. Reimbursement through the national health system emerged as the primary concern, cited by 73.3% (*n* = 11) of participants. The lack of standardization in telehealth practices represented the second most frequent challenge, identified by 53.3% (*n* = 8) of respondents. Additional concerns included limitations in objective examination capabilities and the need for clearer guidelines regarding appropriate telehealth utilization, each reported by 40% (*n* = 6) of participants. The bar plot in Figure 2 provides a visual representation of the participants’ challenges and issues.

## 3. Discussion

This study provides a comprehensive analysis of integrating telemedicine into BoNT-A treatment monitoring for spasticity management. Spasticity, as a chronic condition, demands sustained care and multipronged approaches, and telemedicine is rising to the challenge of addressing these needs, enabling constant patient-physician engagement, which is essential for chronic conditions. Telemedicine directly addresses fundamental challenges in spasticity care by transforming the traditional management approach. The platform enabled physicians to observe patients in their natural environments, leading to more precise assessment of functional limitations and spasticity patterns during daily activities. This contextual understanding proved particularly valuable for post-injection monitoring and treatment optimization, with 73.3% of follow-up assessments successfully conducted remotely. The ability to track BoNT-A treatment responses and make timely adjustments enhanced the precision of therapeutic interventions, while simultaneously reducing the burden of frequent clinical visits for mobility-impaired patients. Many clinicians have only recently begun incorporating telemedicine into their practice, underscoring its emerging role in healthcare. In our study, the majority of clinicians included—approximately 73.3%—reported having less than two years of experience with telemedicine, while only 26.7% had been using it for more than three years. The participants were predominantly physiatrists, and represented a wide range of career stages and clinical experiences, contributing to the diversity and depth of the responses. The gender distribution in our study (60% female) broadly aligned with recent trends in Italian physical medicine and rehabilitation, though exact national statistics for physiatrists are not publicly available.

The first notable finding from the analysis is the participants’ strong appreciation for telemedicine, particularly its effectiveness in streamlining clinical tasks and enhancing time management. Survey results highlighted significant enthusiasm for this mode of healthcare delivery, with many participants showing a high intention to continue using telehealth services. This is a promising indicator for the future integration of telemedicine into routine clinical practice, particularly for spasticity management. These findings align with the existing literature, which consistently reports growing acceptance and satisfaction with telehealth services among healthcare professionals [17,18]. Previous studies, such as Mihai et al. [19], have demonstrated successful implementation of tele-rehabilitation strategies for post-stroke spasticity. In our cohort, physicians reported successful management of treatment modifications in 185 cases (41.1% of teleconsultations), with effectiveness in adjusting exercise programs (210 cases, 46.7%) and monitoring treatment responses.

However, the item assessing whether telemedicine meets patients’ needs received a comparatively lower score, underscoring the need for improvement in two critical areas: fostering more meaningful patient–clinician interactions, and customizing the platform to address the unique needs of each patient. Furthermore, the platform was generally well-perceived, with notable praise for its ease of use and the learning process, corroborated by high TUQ scores for these parameters. This finding aligns with existing research emphasizing the importance of user-friendly telemedicine platforms in facilitating digital transition in healthcare services [20,21,22]. A recent systematic review found that the most influential factors affecting the acceptance of telemedicine among physicians are perceived usefulness, attitude, compatibility, perceived ease of use, self-efficacy, subjective norms, perceived behavioral control, and facilitating condition [23]. Indeed, productivity gains through the use of the system received a moderate score, suggesting that while the platform is easy to learn, it may not directly translate into increased productivity without further refinement or training. 

It is worth noting that despite the general acceptance and satisfaction, telemedicine does not entirely replicate the nuances of face-to-face interaction [24,25]. As our study reveals, lower scores were given for items such as ‘Using the telehealth system, I can see the patient as if we were meeting face-to-face’ and ‘I think the visits provided through the telehealth system are equivalent to face-to-face visits’. This suggests that while telehealth is a valuable tool, there are aspects of patient interaction that need to be optimized to closely mimic in-person visits. Firstly, telemedicine lacks the tactile aspect, meaning clinicians cannot conduct hands-on physical examinations crucial for many medical evaluations. Additionally, issues like poor connectivity, audio-visual lags, or equipment malfunctions can hinder the consultation’s flow. There’s also the challenge of missing subtle non-verbal cues essential for gauging a patient’s discomfort or emotional state. Furthermore, not all patients have the technology access or proficiency to make the most of these services. However, innovations such as artificial intelligence (AI) and other digital tools present opportunities to bridge some of these gaps [26]. AI-driven enhancements can refine audio-visual quality, predict and mitigate connection issues, or even assist clinicians by highlighting important non-verbal cues or changes in a patient’s condition [27,28]. 

While our study did not directly explore patient satisfaction, its significance in healthcare cannot be overlooked. Patient satisfaction offers a window into the perceived quality and effectiveness of care. Generally, physicians perceived a high level of patient satisfaction with the telehealth visits: 66.7% of participants reported that their patients were mostly satisfied, and 13.3% very satisfied. This aligns with the findings of Goldman et al. in their comprehensive study involving 157 patients with various medical conditions, where a significant 79.5% expressed being “very satisfied” with their telehealth experiences. These parallel underscores the broad acceptance and positive reception of telemedicine across different medical contexts and patient populations [29]. 

The versatility of telemedicine in spasticity management is evident from its diverse applications in clinical practice. Clinicians reported using the platform predominantly for follow-up visits, which resonates with the existing literature suggesting telemedicine as an effective tool for maintaining continuity of care, particularly in the context of chronic conditions like spasticity [30,31].

Interestingly, a lower proportion of clinicians reported using telemedicine for initial consultations, which might reflect the perceived limitations in performing comprehensive physical assessments remotely. A total of 53.3% utilized telemedicine for both inspection and functional observation, while 40% employed it for administering evaluation scales or questionnaires. Notably, 26.7% used telemedicine for both environment evaluation and goal definition, highlighting its diverse applications in spasticity management. The virtual environment uniquely positions physicians to dive deeper into treatment evaluation [32]. Through telemedicine, clinicians can meticulously verify aspects of the intervention, from the dosage administered to the precision in targeting the intended muscles. More than that, telemedicine provides a unique lens into the patient’s natural environment, offering insights that might remain obscured within the confines of a clinic. By observing a patient in their familiar surroundings, clinicians gain a richer understanding of the challenges faced and the milestones achieved. This context-specific insight further refines goal-setting, making it more individualized and tailored to the patient’s real-world experiences. In essence, telemedicine does not just facilitate remote consultations; it opens doors to a more comprehensive, context-aware evaluation and a deeper collaborative involvement in the rehabilitative journey post BoNT-A treatment.

The evolution of healthcare in the digital age necessitates a holistic approach that seamlessly merges the strengths of both telemedicine and traditional in-person visits. This study revealed that while telemedicine excels in monitoring treatment responses and adjusting therapeutic programs, certain clinical scenarios require physical presence, notably initial BoNT-A administration and complex physical assessments.

A hybrid healthcare model, which alternates between these two methods, offers a pragmatic solution tailored to the diverse and dynamic needs of patients. While telemedicine boasts undeniable benefits in terms of accessibility, cost-effectiveness, and convenience, there remain clinical scenarios where the physical presence of a patient is imperative. Evaluations derived from a hands-on physical examination or specialized interventions such as BoNT-A injections cannot be replicated in a virtual environment. However, for follow-ups, discussions, or consultations that do not necessitate direct physical interaction, telemedicine can provide an efficient alternative, alleviating the logistical and financial burdens often associated with in-person visits. Patient circumstances and clinical requirements naturally guide the selection between telemedicine and in-person consultations. Factors such as distance from treatment centers, mobility limitations, and complexity of assessment needs influence this decision. The success of integrating both modalities depends on clear clinical protocols and maintaining consistent care quality across both formats. Transitioning to such a hybrid model not only addresses the immediate concerns of patient care but also anticipates and adapts to future healthcare challenges, ensuring continuity, adaptability, and the highest standard of care. The adoption of a hybrid healthcare model is not without precedent, as it is already being successfully implemented in various other medical disciplines [33,34,35,36]. This blended approach could prove particularly beneficial for spasticity management, where the intricate balance between comprehensive assessment and patient convenience is crucial. Integrating both in-person and telemedicine visits would enable clinicians to tailor their approach based on the immediate needs of spasticity patients, ensuring effective and responsive care. 

Despite the overall positive response, clinicians highlighted several challenges that need to be addressed to fully integrate telemedicine into routine clinical practice. One major concern was the reimbursement of telemedicine visits by the national health system, reflecting broader debates about sustainable financial models for telehealth services. While telemedicine services are officially reimbursable under the Italian national health system through a payment parity mechanism (ensuring providers receive fixed amounts regardless of service delivery mode), practical implementation remains challenging. Regional variations in policies and incomplete pricing protocols, particularly for device-connected services like the Maia platform, create uncertainty in reimbursement processes. The lack of clear policies and standardized reimbursement frameworks creates uncertainty and may hinder the widespread adoption of telemedicine, particularly in resource-constrained settings. Another significant issue identified was the absence of standardization in telemedicine practices. Clinicians emphasized the need for uniform protocols and guidelines to ensure consistent and effective care delivery across different platforms and settings. The inability to perform comprehensive objective examinations was also a notable limitation, particularly for conditions like spasticity, which often require detailed physical assessments. This gap underscores the need for developing and integrating advanced tools, such as remote diagnostic technologies or hybrid care models, to complement telehealth services.

This study had several limitations. The number of participants was relatively small and limited to clinicians specializing in spasticity, which may restrict the generalizability of the findings. The small sample size could also affect the robustness of the reliability analysis, so the results should be interpreted with caution. Furthermore, the findings are specifically situated within the Italian National Health Service framework, which may differ significantly from other healthcare systems in terms of reimbursement structures, professional practice regulations, and technology infrastructure requirements. Cultural considerations also play a crucial role, as the acceptance and implementation of telemedicine may be influenced by local medical practice patterns, patient preferences and expectations, and healthcare provider training and familiarity with technology. Future studies should examine the transferability of our findings to different healthcare systems and cultural contexts. Moreover, a more diverse sample, encompassing clinicians from various specialties, to provide broader insights, should be considered. Additionally, this study did not explore patients’ perspectives, which are essential for optimizing telemedicine platforms. The subjective nature of the TUQ also presents a limitation, as it may be influenced by response bias. Future studies could benefit from adopting a longitudinal design to evaluate how clinician and patient satisfaction with telemedicine evolves over time with sustained use.

## 4. Conclusions

This study underscores the potential of a telemedicine platforms in the management of spasticity while simultaneously highlighting areas that need attention. By addressing these areas, telemedicine could serve as a powerful adjunct to traditional healthcare services, fostering greater accessibility, continuity, and patient-centeredness in spasticity management. Future research directions should encompass studies that further explore the identified challenges and assess the impact of telemedicine on patient outcomes in spasticity management.

## 5. Materials and Methods

A multicenter cross-sectional survey was conducted from March 2023 to June 2023. The participants in this study were Italian physicians who routinely manage patients with spasticity. All respondents were using the Maia Connected Care telemedicine platform (AB Medica Spa, Cerro Maggiore, MI, Italy) exclusively for teleconsultation in their practices. Teleconsultation, one the services offered as part of telemedicine, involves a therapeutic or medical act carried out remotely between a health professional and a patient [37]. The selection of participants followed strict eligibility criteria. Participating centers were required to have served as beta testers for the Maia Platform for a minimum of one year. Individual clinicians needed at least six months of experience using the Maia Platform and to have maintained active practice in spasticity management using BoNT-A. Given the survey’s administration in English, participants were required to demonstrate fluency in the English language.

The selection process began with the identification of twelve eligible centers across Italy selected from a list provided by AB Medica Spa (Cerro Maggiore, MI, Italy). Among these centers, two declined participation citing resource constraints, while two additional centers were excluded due to insufficient platform experience (less than one year of systematic use). The final sample included eight participating centers. From these centers, eighteen eligible physicians were initially identified. Two physicians declined participation due to time constraints, and one provided incomplete responses, resulting in the final sample of fifteen participants.

To ensure a robust analysis, our usability study involved 15 participants. This decision was informed by Jakob Nielsen’s 1994 recommendation, which suggests that testing with five users is typically sufficient to identify approximately 85% of usability issues [38]. However, Faulkner demonstrated that while five users might suffice for basic testing, larger sample sizes—particularly 10–20 users—provide more reliable results and reduce the risk of missing critical usability issues, especially when dealing with complex interactions or diverse user groups [39]. Furthermore, a priori power analysis was conducted using G*Power 3.1.9.7 to determine the required sample size for detecting significant effects in usability evaluation. For the primary analysis of system usability, we set α = 0.05, power (1 − β) = 0.80, and anticipated a medium-to-large effect size (d = 0.8) based on previous telemedicine usability studies. This analysis indicated a minimum required sample size of 12 participants. Our final sample of 15 participants exceeded this requirement, achieving an actual power of 0.89. The reliability analysis of the modified TUQ required additional sample size considerations. Using a null hypothesis of α_0_ = 0.60 (minimally acceptable reliability) versus α_1_ = 0.84 (expected reliability based on pilot data), with 21 items, β = 0.20, and α = 0.05, calculations indicated a minimum required sample size of 15 participants.

### 5.1. Telemedicine Platform: Maia Connected Care

Maia Connected Care (AB Medica Spa, Italy) represents a Class IIA medical device engineered for comprehensive patient care management. The platform operates on a cloud-based software infrastructure with servers located exclusively in Italy. The system demonstrates full GDPR (General Data Protection Regulation) compliance and incorporates extensive interface capabilities with various medical devices for vital parameter monitoring. An integrated clinical activity management system forms the backbone of the platform’s functionality. The Maia Connected Care platform supports both video and voice-only consultations, though video capability was used in all cases in this study to enable visual assessment of movement patterns and spasticity. The platform implements robust security measures, including end-to-end encryption for all communications and a secure server infrastructure maintained within Italian territory. Regular security audits and updates ensure ongoing protection of sensitive data. The system maintains strict compliance with European medical device regulations and handles protected health information according to GDPR guidelines.

Detailed information about the telemedicine service provided by AB Medica, including technical requirements, patient privacy policies, and user guidelines, will be provided under request.

### 5.2. Survey

The comprehensive survey instrument was divided into four distinct sections, each designed to capture specific aspects of telemedicine implementation in spasticity management.

Section 1 focused on demographics and professional background, gathering information about age, gender, specialty, and years of experience. This section also explored the various modalities through which telehealth services were provided and specific patterns of technology utilization in clinical practice.

Section 2 incorporated the modified Telemedicine Usability Questionnaire (TUQ), consisting of 21 items distributed across six domains. The questionnaire employed a seven-point Likert scale ranging from 1 (“Strongly Disagree”) to 7 (“Strongly Agree”). The domains encompassed usefulness, ease of use, interface quality, interaction quality, reliability, and satisfaction. The modification process involved adapting the original TUQ for the specific context of spasticity management, with particular attention catered to BoNT-A treatment protocols. In line with the initial validation study [40], which allows for adaptation to different contexts, we made specific modifications to tailor the questionnaire to our focus on spasticity management using the Maia Connected Care telemedicine platform. Questions were carefully adjusted to reflect the healthcare provider perspective. Reliability assessment of the modified instrument yielded a Cronbach’s alpha of 0.84 for original items and 0.87 for standardized items, indicating strong internal consistency. The sample included 15 valid responses, with no exclusions, confirming the stability and dependability of the TUQ in this context.

Section 3 included questions to evaluate physicians’ perceptions of patient satisfaction, the reliability of rehabilitation goal-setting, and the effectiveness of post-infiltration assessments conducted via telemedicine. Responses to these questions were captured using the Likert scale (1–7 range). The inclusion of this section sheds light on the practicality and utility of telemedicine in the specific and critical aspect of managing spasticity, especially in patients undergoing BoNT-A treatments.

Lastly, Section 4 of the survey was designed to identify potential barriers to the broader adoption of telehealth services. This section incorporated a series of dichotomous (yes/no) questions addressing potential challenges like technical issues, logistical concerns, patient safety, quality of care, and legal or regulatory barriers.

Data collection proceeded through a structured digital platform utilizing Google Forms. Prior to participation, digital informed consent was obtained from all participants, with explicit verification of English language proficiency. Technical support remained available throughout the survey completion process to address any potential issues. A response verification process was implemented to ensure data quality and completeness.

### 5.3. Statistical Analysis

Following the collection of data, a comprehensive statistical analysis was conducted to interpret the results. To facilitate the gathering of this data, Google Forms was used for administering the questionnaire to the clinicians. Descriptive statistics were used to summarize and describe the basic features of the data obtained from the TUQ. This included measures of mean (standard deviation) for each question on the TUQ. For categorical data, frequency distributions were calculated. This involved counting the number of cases that fell into each category for the various categorical variables in the TUQ. The results were then presented in the form of frequency tables, bar charts, and pie charts, providing a visual representation of the data. The analysis was performed using the Statistical Package for the Social Sciences (SPSS) version 26.

## Figures and Tables

**Table 1:** Demographic and professional characteristics of participants.

***n* = 15 participants**	*n* (%)
**Gender**	
Male	6 (40%)
Female	9 (60%)
**Mean age** years (SD)	45 (10.26)
**Telemedicine experience**	
<1 year	3 (20%)
1–2 years	8 (53.3%)
>3 years	4 (26.7%)
**Type of teleconsultation**	
First visit	4 (26.6%)
Follow up	15 (100%)
On demand visit	6 (40%)
**Examination performed**	
Inspection	8 (53.3%)
Functional observation	8 (53.3%)
Environment evaluation	4 (26.7%)
Administration of evaluation scales and/or questionnaires	6 (40%)
Goal definition	4 (26.7%)
**Scales used**	
VAS/NRS	14
Modified Ashworth Scale	3 (20%)
Scale of independence (e.g., Barthel Index)	5 (33.3%)
Quality of life questionnaire	3 (20%)

**Table 2:** Detailed scores from the Telemedicine Usability Questionnaire express as mean (standard deviation, SD).

TUQ Item	Mean (SD)
**Usefulness**	**16.07 (2.15)**
Telehealth improves the services I can offer	5.87 (0.99)
Telehealth saves me time during my work	5.27 (0.59)
Telehealth provides for patients needs	4.93 (1.10)
**Ease of use and learnability**	**17.87 (1.73)**
It was simple to use this telehealth system	6.40 (0.63)
It was easy to learn this telehealth system	6.47 (0.74)
I believe I could become more productive using this telehealth system	5.00 (0.76)
**Interface Quality**	**23.13 (2.35)**
The interaction with this telehealth system is pleasant	5.80 (0.77)
I like using this telehealth system	5.84 (0.74)
This system is simple and easy to understand	6.40 (0.63)
This system could do everything I would want it to be able to do	5.07 (1.16)
**Interaction Quality**	**21.60 (2.23)**
I could easily talk with the patient using the telehealth system	5.87 (0.64)
I could clearly hear the patient using the telehealth system	5.93 (0.70)
I felt I was able to express myself effectively using the telehealth system	6.07 (0.80)
Using the telehealth system, I can see the patient as well as if we met in person	3.73 (1.44)
**Reliability**	**13.07 (2.60)**
I think the visits provided through the telehealth system are the same as in-person visits	3.13 (1.30)
Whenever I made a mistake using the service, I could recover it easily and quickly	5.07 (0.80)
The system provided clear error messages that clearly guide me how to fix the problems	4.87 (1.19)
**Satisfaction and future use**	**24.47 (2.42)**
I felt comfortable communicating with the patient using the telehealth system	6.00 (0.93)
Telehealth is an acceptable way to manage patients with spasticity	5.67 (0.90)
I would use telehealth services again	6.47 (0.52)
Overall, I am satisfied with this telehealth system	6.33 (0.62)

## Data Availability

The original contributions presented in this study are included in this article. Further inquiries can be directed to the corresponding authors.

## References

[1] Sood S., Mbarika V., Jugoo S., Dookhy R., Doarn C.R., Prakash N., Merrell R.C. (2007). What Is Telemedicine? A Collection of 104 Peer-Reviewed Perspectives and Theoretical Underpinnings. Telemed. e-Health.

[2] Hsiao V., Chandereng T., Huebner J.A., Kunstman D.T., Flood G.E., Tevaarwerk A.J., Schneider D.F. (2023). Telemedicine Use across Medical Specialties and Diagnoses. Appl. Clin. Inform..

[3] Mariani M.V., Pierucci N., Forleo G.B., Schiavone M., Bernardini A., Gasperetti A., Mitacchione G., Mei M., Giunta G., Piro A. (2023). The Feasibility, Effectiveness and Acceptance of Virtual Visits as Compared to In-Person Visits among Clinical Electrophysiology Patients during the COVID-19 Pandemic. J. Clin. Med..

[4] Dorsey E.R., Topol E.J. (2016). State of Telehealth. N. Engl. J. Med..

[5] Tenforde A.S., Hefner J.E., Kodish-Wachs J.E., Iaccarino M.A., Paganoni S. (2017). Telehealth in Physical Medicine and Rehabilitation: A Narrative Review. PM R.

[6] Lance J.W. (1980). The Control of Muscle Tone, Reflexes, and Movement: Robert Wartenberg Lecture. Neurology.

[7] Esquenazi A. (2011). The Human and Economic Burden of Poststroke Spasticity and Muscle Overactivity. J. Clin. Outcomes Manag..

[8] Sheean G., McGuire J.R. (2009). Spastic Hypertonia and Movement Disorders: Pathophysiology, Clinical Presentation, and Quantification. PM R.

[9] Simpson D.M., Hallett M., Ashman E.J., Comella C.L., Green M.W., Gronseth G.S., Armstrong M.J., Gloss D., Potrebic S., Jankovic J. (2016). Practice Guideline Update Summary: Botulinum Neurotoxin for the Treatment of Blepharospasm, Cervical Dystonia, Adult Spasticity, and Headache: Report of the Guideline Development Subcommittee of the American Academy of Neurology. Neurology.

[10] Vidal-Alaball J., Acosta-Roja R., PastorHernández N., Luque U.S., Morrison D., Pérez S.N., Perez-Llano J., Vèrges A.S., Seguí F.L. (2020). Telemedicine in the Face of the COVID-19 Pandemic. Aten. Primaria.

[11] Harper K.A., Butler E.C., Hacker M.L., Naik A., Eoff B.R., Phibbs F.T., Isaacs D.A., Gallion S.J., Thomas E.P., Scott J.L. (2021). A Comparative Evaluation of Telehealth and Direct Assessment When Screening for Spasticity in Residents of Two Long-Term Care Facilities. Clin. Rehabil..

[12] Verduzco-Gutierrez M., Romanoski N.L., Capizzi A.N., Reebye R.N., Kotteduwa Jayawarden S., Ketchum N.C., O’Dell M. (2020). Spasticity Outpatient Evaluation via Telemedicine: A Practical Framework. Am. J. Phys. Med. Rehabil..

[13] Ross J., Stevenson F., Lau R., Murray E. (2016). Factors That Influence the Implementation of E-Health: A Systematic Review of Systematic Reviews (an Update). Implement. Sci..

[14] Ricci G., Caraffa A.M., Gibelli F. (2023). Telemedicine as a Strategic Tool to Enhance the Effectiveness of Care Processes: Technological and Regulatory Evolution over the Past Two Decades. Healthcare.

[15] Parretti C., La Regina M., Tortu C., Candido G., Tartaglia R. (2023). Telemedicine in Italy, the Starting Point. Intern. Emerg. Med..

[16] Ferorelli D., Nardelli L., Spagnolo L., Corradi S., Silvestre M., Misceo F., Marrone M., Zotti F., Mandarelli G., Solarino B. (2020). Medical Legal Aspects of Telemedicine in Italy: Application Fields, Professional Liability and Focus on Care Services During the COVID-19 Health Emergency. J. Prim. Care Community Health.

[17] Hoff T., Lee D.R. (2022). Physician Satisfaction With Telehealth: A Systematic Review and Agenda for Future Research. Qual. Manag. Health Care.

[18] Choi J.S., Lin M., Park S., Abdur-Rahman F., Kim J.H., Voelker C.C.J. (2022). Physician Satisfaction with Telemedicine and In-Person Visits in Otolaryngology. Am. J. Otolaryngol.—Head Neck Med. Surg..

[19] Mihai E.E., Popescu M.N., Beiu C., Gheorghe L., Berteanu M. (2021). Tele-Rehabilitation Strategies for a Patient With Post-Stroke Spasticity: A Powerful Tool Amid the COVID-19 Pandemic. Cureus.

[20] Fouquet S.D., Miranda A.T. (2020). Asking the Right Questions—Human Factors Considerations for Telemedicine Design. Curr. Allergy Asthma Rep..

[21] Bunnell B.E., Barrera J.F., Paige S.R., Turner D., Welch B.M. (2020). Acceptability of Telemedicine Features to Promote Its Uptake in Practice: A Survey of Community Telemental Health Providers. Int. J. Environ. Res. Public Health.

[22] Yellowlees P.M. (2005). Successfully Developing a Telemedicine System. J. Telemed. Telecare.

[23] Garavand A., Aslani N., Nadri H., Abedini S., Dehghan S. (2022). Acceptance of Telemedicine Technology among Physicians: A Systematic Review. Inform. Med. Unlocked.

[24] Donaghy E., Atherton H., Hammersley V., McNeilly H., Bikker A., Robbins L., Campbell J., McKinstry B. (2019). Acceptability, Benefits, and Challenges of Video Consulting: A Qualitative Study in Primary Care. Br. J. General. Pract..

[25] Cheshire W.P., Barrett K.M., Eidelman B.H., Mauricio E.A., Huang J.F., Freeman W.D., Robinson M.T., Salomon G.R., Ball C.T., Gamble D.M. (2021). Patient Perception of Physician Empathy in Stroke Telemedicine. J. Telemed. Telecare.

[26] Vittoradolfo T., Abbas S., Cingolani M. (2023). Artificial Intelligence and Digital Medicine for Integrated Home Care Services in Italy: Opportunities and Limits. Front. Public Health.

[27] Kuziemsky C., Maeder A.J., John O., Gogia S.B., Basu A., Meher S., Ito M. (2019). Role of Artificial Intelligence within the Telehealth Domain. Yearb. Med. Inform..

[28] Javaid M., Haleem A., Pratap Singh R., Suman R., Rab S. (2022). Significance of Machine Learning in Healthcare: Features, Pillars and Applications. Int. J. Intell. Netw..

[29] Goldman J.G., Merkitch D., Brewington D., Peirce H., Rho M., Jayabalan P., Curran J., Brennan K. (2023). Patient Experiences Receiving Rehabilitation Care via Telehealth: Identifying Opportunities for Remote Care. Front. Rehabil. Sci..

[30] Ma Y., Zhao C., Zhao Y., Lu J., Jiang H., Cao Y., Xu Y. (2022). Telemedicine Application in Patients with Chronic Disease: A Systematic Review and Meta-Analysis. BMC Med. Inform. Decis. Mak..

[31] Noel K., Ellison B. (2020). Inclusive Innovation in Telehealth. NPJ Digit. Med..

[32] Gomez T., Anaya Y.B., Shih K.J., Tarn D.M. (2021). A Qualitative Study of Primary Care Physicians’ Experiences with Telemedicine during COVID-19. J. Am. Board. Fam. Med..

[33] Patton E.W., Saia K., Stein M.D. (2021). Integrated Substance Use and Prenatal Care Delivery in the Era of COVID-19. J. Subst. Abus. Treat..

[34] Cascella M., Schiavo D., Grizzuti M., Cristina Romano M., Coluccia S., Bimonte S., Cuomo A. (2023). Implementation of a Hybrid Care Model for Telemedicine-Based Cancer Pain Management at the Cancer Center of Naples, Italy: A Cohort Study. In Vivo.

[35] Toschi E., Adam A., Hurlbert R., Frimpong N., Slyne C., Laffel L.M., Munshi M. (2023). 361-OR: Hybrid Care Model Combining Telemedicine and Office Visits for Diabetes Management Is Effective in Older Adults with Type 1 Diabetes (T1D) Using Continuous Glucose Monitors (CGM). Diabetes.

[36] Chen M., Mohd Said N., Mohd Rais N.C., Ho F., Ling N., Chun M., Ng Y.S., Eng W.N., Yao Y., Korc-Grodzicki B. (2022). Remaining Agile in the COVID-19 Pandemic Healthcare Landscape—How We Adopted a Hybrid Telemedicine Geriatric Oncology Care Model in an Academic Tertiary Cancer Center. J. Geriatr. Oncol..

[37] Staccini P., Daniel C., Dart T., Bouhaddou O. (2014). Medical Informatics, e-Health Fundamentals and Applications.

[38] Nielsen J. (1993). Usability Heuristics. Usability Engineering.

[39] Faulkner L. (2003). Beyond the Five-User Assumption: Benefits of Increased Sample Sizes in Usability Testing. Behav. Res. Methods Instrum. Comput..

[40] Parmanto B., Lewis A.N., Graham K.M., Bertolet M.H. (2016). Development of the Telehealth Usability Questionnaire (TUQ). Int. J. Telerehabilit..

